# The effect of locally delivered recombinant human bone morphogenetic protein-2 with hydroxyapatite/tri-calcium phosphate on the biomechanical properties of bone in diabetes-related osteoporosis

**DOI:** 10.1007/s10195-014-0327-6

**Published:** 2014-11-25

**Authors:** Frank A. Liporace, Eric A. Breitbart, Richard S. Yoon, Erin Doyle, David N. Paglia, Sheldon Lin

**Affiliations:** 1 Division of Orthopaedic Trauma, Department of Orthopaedic Surgery, NYU Hospital for Joint Diseases, 301 E 17th Street, Suite 1402, New York, NY 10003 USA; 2 Division of Orthopaedic Trauma, Department of Orthopaedic Trauma, UMDNJ, New Jersey Medical School, Newark, NJ 07101 USA

**Keywords:** rhBMP-2, Calcium phosphate, Hydroxyapatite, Diabetes, Fracture, Osteoporosis

## Abstract

**Background:**

Recombinant human bone morphogenetic protein-2 (rhBMP-2) is particularly effective in improving osteogenesis in patients with diminished bone healing capabilities, such as individuals with type 1 diabetes mellitus (T1DM) who have impaired bone healing capabilities and increased risk of developing osteoporosis. This study measured the effects of rhBMP-2 treatment on osteogenesis by observing the dose-dependent effect of localized delivery of rhBMP-2 on biomechanical parameters of bone using a hydroxyapatite/tri-calcium phosphate (HA/TCP) carrier in a T1DM-related osteoporosis animal model.

**Materials and methods:**

Two different doses of rhBMP-2 (LD low dose, HD high dose) with a HA/TCP carrier were injected into the femoral intramedullary canal of rats with T1DM-related osteoporosis. Two more diabetic rat groups were injected with saline alone and with HA/TCP carrier alone. Radiographs and micro-computed tomography were utilized for qualitative assessment of bone mineral density (BMD). Biomechanical testing occurred at 4- and 8-week time points; parameters tested included torque to failure, torsional rigidity, shear stress, and shear modulus.

**Results:**

At the 4-week time point, the LD and HD groups both exhibited significantly higher BMD than controls; at the 8-week time point, the HD group exhibited significantly higher BMD than controls. Biomechanical testing revealed dose-dependent, higher trends in all parameters tested at the 4- and 8-week time points, with minimal significant differences.

**Conclusions:**

Groups treated with rhBMP-2 demonstrated improved bone mineral density at both 4 and 8 weeks compared to control saline groups, in addition to strong trends towards improvement of intrinsic and extrinsic biomechanical properties when compared to control groups. Data revealed trends toward dose-dependent increases in peak torque, torsional rigidity, shear stress, and shear modulus 4 weeks after rhBMP-2 treatment.

**Level of evidence:**

Not applicable.

## Introduction

Diabetes mellitus is a devastating and life-altering disease, affecting over 20 million people in the USA [1]. Ten percent of those 20 million people suffer from type 1 diabetes (T1DM), in which a 20 % prevalence of osteoporosis with significant bone loss has been reported when compared to healthy, age-matched subjects. Multiple T1DM animal models have been used to further observe and understand T1DM-related osteoporosis. Related animal models have exhibited weakened and altered bony architecture, with potential causes secondary to lack of vascular supply, and osteocalcin receptor malfunction [2–4].

With a clear clinical need to identify targets to combat T1DM-related osteoporosis, bone morphogenetic proteins (BMPs) have become an obvious research target [5]. BMPs are important in bone metabolism and exert their effects on bone through the tightly coupled processes of osteoclastogenesis and osteoblastogenesis [6–8]. The BMP family, in addition to being the largest member of the transforming growth factor (TGF)-β superfamily, is also involved in almost all processes related to skeletal development, morphogenesis, angiogenesis, and homeostasis [9–12]. Because of BMPs’ essential role in bone regeneration, several delivery models have been studied, including the utilization of hydroxyapatite/tri-calcium phosphate (HA/TCP) as a primary delivery platform and scaffold [13–18]. With the frequent use of HA/TCP in the fragility fracture setting, this is an obvious delivery model and early results as an osteogenic promoter have been promising [15, 18].

Given the prevalence of T1DM-related osteoporosis and the lack of safe and effective anabolic therapies, studying a corollary between BMP-2 and its possible effect on T1DM-related osteoporosis seems like a natural progression to a potentially beneficial clinical modality [19]. This study evaluated the dose-dependent effect of local rhBMP-2 on biomechanical parameters of bone in the T1DM-related osteoporotic state by combining its possible improved efficacy with a HA/TCP delivery carrier, and using the spontaneous diabetic BB Wistar rat model.

## Materials and methods

### Animals, preoperative preparation, and cohort formulation

Male BB Wistar rats were used in this study; these rats develop T1DM through an autoimmune, selective destruction of the pancreatic beta cells. The BB Wistar rat currently represents a close homology of human T1DM in a laboratory animal [20]. The BB Wistar rats were obtained from a breeding colony established at the senior author’s institution with breeding pairs obtained from BioBreeding (Toronto, Canada). The Institutional Animal Care and Use Committee (IACUC) approved all research protocols. The diabetes-prone BB Wistar rats develop diabetes at an incidence rate of approximately 30–45 % at 70–120 days of age. Urine from the diabetic BB Wistar rats was checked for glycosuria three times a week. Once glycosuria was detected, blood specimens obtained from tail veins were tested for blood glucose levels greater than 250 mg/dl. If the reading exceeded this value, an insulin implant (Linplant^®^) which provided constant insulin release for approximately 30 days was aseptically placed subcutaneously in the dorsal neck. Blood glucose levels were determined by the glucose-oxidase method (Accu-Chek Advantage, Roche Diagnostics, Indianapolis, IN, USA) obtained through the posterior dorsal tail vein. The diabetic rats were treated with insulin to maintain blood glucose levels between 300 and 450 mg/dl, representing a poorly controlled state of diabetes with glycosuria but no sign of ketonuria. All animals were evaluated for blood glucose levels in the first 24 h after insulin implantation. If the desired blood glucose level was not achieved, an additional amount of insulin implant was given to achieve the appropriate level. The blood glucose levels were evaluated three times a week for the appropriate level of blood glucose control and animals were treated accordingly. Using the model based on Verhaeghe et al. [21], animals were considered osteoporotic 12 weeks after onset of diabetes. Surgery was performed 12 weeks after onset of diabetes in all groups.

All groups except for the saline group required preparation of the HA/TCP carrier for injection. The procedure is as follows: first, 0.2 g HA/TCP (particle size <0.53 µm) was mixed with 100 µl of the appropriate rhBMP-2 dose. The mixture was allowed to stand for 15 min to allow the rhBMP-2 to bind to the HA/TCP carrier. After 15 min, 100 µl of buffer solution was added and mixed. The mixture was then drawn into a 1-ml syringe ready for injection into the femoral canal. Note that the HA/TCP control group received buffer solution twice in place of rhBMP-2. All groups received approximately 0.1 ml injections into the femur.

A surgical procedure was performed in which injections of varying doses of BMP-2, saline, and HA/TCP carrier (particle size <0.53 µm) were injected into the intramedullary (IM) canal of the femur. There were four different treatment groups:

A. diabetic w/saline
B. diabetic w/HA/TCP carrier
C. diabetic w/HA/TCP carrier + rhBMP-2 Low Dose: 0.11 mg/ml (approximate actual dose applied: 2.75 μg per femur)
D. diabetic w/HA/TCP carrier + rhBMP-2 High Dose: 0.22 mg/ml (approximate actual dose applied: 5.5 μg per femur)

Group A consisted of diabetes-related osteoporotic rats receiving an intermedullary injection of saline to determine whether the reaming of the IM canal had any effect. Group B consisted of diabetes-related osteoporotic rats receiving an IM injection of the HA/TCP carrier alone to determine whether there is a carrier-dependent effect. Groups C and D consisted of diabetes-related osteoporotic rats that received IM injections of the HA/TCP carrier with two different doses of rhBMP-2: 0.11 mg/ml for the low dose (LD) and 0.22 mg/ml for the high dose (HD). All groups received approximately 0.1 ml injection of the appropriate treatment into the IM canal of the femur.

The study cohorts consisted of 48 rats divided into four different experimental groups at both 4- and 8-week time-points, with *n* = 6 per group (Table 1). Losses included death from anesthesia given for Linplant insulin treatment, which occurred with two animals (accounting for two groups with *n* = 5). The 8-week saline group had 3 extra (*n* = 8) animals, as they were available for surgery before rhBMP-2 was obtained.

**Table 1:** Experimental groups

Groups	Testing time-point	
	4 weeks	8 weeks
Diabetic + saline	5 rats	8 rats
Diabetic + HA/TCP + buffer	6 rats	6 rats
Diabetic + HA/TCP + low-dose rhBMP-2	5 rats	6 rats
Diabetic + HA/TCP + high-dose rhBMP-2	6 rats	6 rats

### Surgical method

The surgical procedure for the rhBMP-2 treatment in the diabetes-related osteoporotic model is as follows: general anesthesia was administered by intraperitoneal injection of ketamine (60 mg/kg) and xylazine (8 mg/kg). After adequate anesthesia, each rat was shaved and prepped with Betadine^®^ and 70 % alcohol. A 4-mm longitudinal skin incision was made over the patella. The patella was then dislocated laterally and the interchondylar notch of the distal femur was exposed. An entry hole was made with an 18-gauge needle and the IM canal was then reamed. At this time the appropriate treatment was administered by injecting either saline, HA/TCP carrier, low-dose BMP-2 and HA/TCP or high-dose BMP-2 and HA/TCP into the IM canal followed by wound closure with a 4-0 vicryl. The rats were allowed to ambulate freely post-surgery.

### Postoperative evaluation

Radiographs were taken immediately after surgery to ensure that the IM canal was properly entered without damage to the femur as well as to detect the presence of material in the canal. Radiographs were taken using a Model 804 Faxitron (Field Emission Corp., McMinnville, OR, USA) and 8 × 10 Kodak Min-R 2000 mammography film (Eastman Kodak Co., Rochester, NY, USA) with an exposure time of 30 s at 55 peak kilovoltage (kVp). Subsequent radiographs were taken at 2-week intervals to observe any effect changes in the bone as well as to check for infection. The animals were killed and both right and left femurs were resected, and magnified radiographs of both anterior–posterior (AP) and lateral (L) views were taken. Radiographs were not used for quantification but were used for qualitative observation. Micro-computed tomography (CT) imaging studies were performed on all samples in order to provide a higher quality imaging study for additional qualitative evaluation to calculate BMD.

Animals were killed at 4 and 8 weeks post-surgery. The treated femora as well as the contralateral femora were resected and stored at −20 °C in saline (0.9 % NaCl)-soaked gauze. Prior to testing, all femora were removed from the freezer and allowed to thaw to room temperature. Samples were cleaned of soft tissue and femur dimensions were measured using calipers. Proximal and distal ends of the femora were then embedded in ¾-inch (6-mm) square nuts with Wood’s metal (Alfa Aesar, Ward Hill, MA, USA), leaving an approximate gauge length of 14 mm. The samples were then torsionally tested to failure using a servo hydraulics machine (MTS Systems Corp., Eden Prairie, MN, USA) with a 20 N m torque cell (Interface, Scotsdale, AZ, USA) at a rate of 2.0°/s. Maximum torque to failure and angle of failure were obtained from the torque force to angular displacement data. Standard equations modeling each femur as a hollow ellipse were used to calculate polar moment of inertia. Peak torque to failure (*T*_max_), angle of failure (*θ*), gauge length (*L*), and polar moment of inertia (*J*) were used to calculate maximum torsional rigidity (TR), shear modulus (SM), and maximum torsional shear stress (SS). Mechanical testing values were normalized to minimize inter-animal variability. Normalized data is presented as a percentage and was calculated by dividing treated femur values by the contralateral untreated femur values.

### Statistical analysis

Data was analyzed using the Sigma Stat (version 3.0) statistical analysis program. Analysis was performed using one-way ANOVA at each time point (4 and 8 weeks) for all four treatment groups. A 2-sample *t* test was used to compare combined LD and HD rhBMP-2 groups against combined control groups. In all cases significance was determined as a *p* value of less than 0.05. Power analysis was performed using the Pass power analysis software (6.0, NCSS, Kaysville, UT, USA).

## Results

Inclusion of animals in this study was based upon 12 weeks duration of poorly controlled T1DM in the spontaneous diabetic BB Wistar rat, at which point the animal was considered osteoporotic. Average blood glucose levels, age at surgery, weight at surgery, weight at death, and weight change for all four treatment groups showed no statistical differences when compared within their respective time points (Table 2).

**Table 2:** General health at 4- and 8-week time-points

Groups	Blood glucose* (mg/dl)	Age at surgery (days)	Weight at surgery (g)	Weight at death (g)	Weight change (g)
Saline (*n* = 5)					
4 weeks	444 ± 40	182 ± 15	408 ± 36	404 ± 28	−4 ± 17
8 weeks	421 ± 44	184 ± 11	369 ± 30	401 ± 30	33 ± 24
Buffer + HA/TCP (*n* = 6)					
4 weeks	493 ± 22	187 ± 12	398 ± 40	385 ± 45	−13 ± 43
8 weeks	433 ± 14	175 ± 20	394 ± 33	404 ± 35	10 ± 16
Low-dose rhBMP-2 + HA/TCP (*n* = 5)					
4 weeks	478 ± 38	174 ± 10	401 ± 15	407 ± 18	6 ± 7
8 weeks	440 ± 37	161 ± 8	383 ± 60	383 ± 60	2 ± 17
High-dose rhBMP-2+ HA/TCP (*n* = 6)					
4 weeks	474 ± 44	176 ± 13	404 ± 27	387 ± 32	−16 ± 22
8 weeks	430 ± 27	177 ± 13	425 ± 23	416 ± 11	−8 ± 18

The data represent average values ± standard deviation

* Blood glucose data represents mean data from animals in each treatment group resulting from measurements taken from blood drawn two or three times per week from each animal from onset of T1DM up to death

**Fig. 1:**
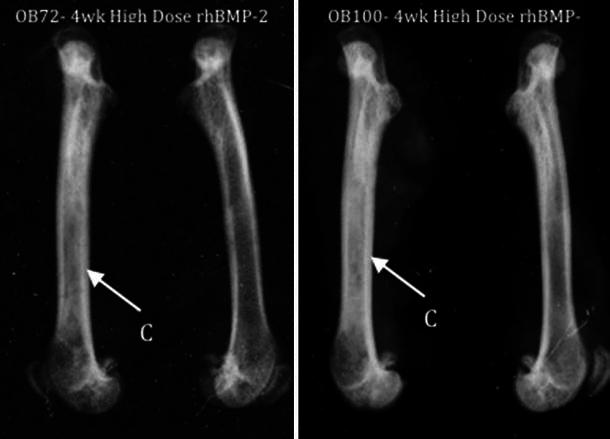
Magnified lateral radiographs of re-sected femora at 4 weeks. *C* Qualitative observation of cortical thickening in rhBMP-2 treated femurs. Note: treated (right) femurs appear on the *left* in both radiographic images

**Fig. 2:**
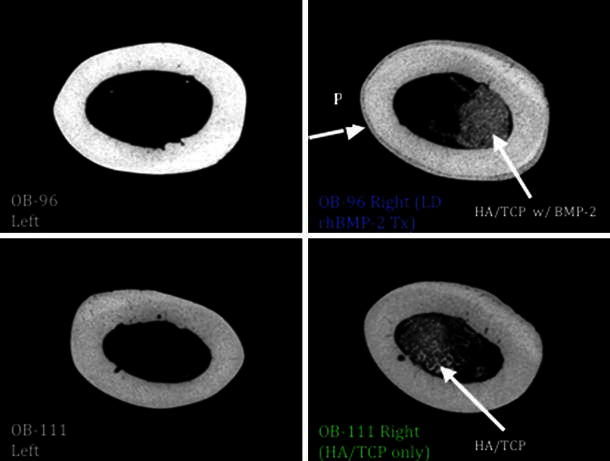
MicroCT midshaft cortical scan: note new periosteal bone growth (*P*) in the LD rhBMP-2 treated femur on the *top right* compared to contralateral limb and HA/TCP-only treated sample

Radiographic images were analyzed independently by two blinded reviewers, who were asked to compare the thickness of the cortices of left (untreated) and right (treated) femurs. Qualitative analysis revealed cortical thickening in the diaphysis in over 75 % of the femurs (18/23) treated with rhBMP-2 in both the LD (9/11) and HD (9/12) groups (Fig. 1) compared to the untreated femur, significantly higher than in the saline control (4/13) and HA/TCP control (5/12) groups (*p* < 0.01). To supplement these plain radiographs, microCT was performed, with similar observations noted amongst the experimental groups (Fig. 2).

In regards to bone mineral density, at the 4-week time point a significant improvement in BMD was seen in both the LD and HD rhBMP-2 groups compared to the saline control (*p* = 0.007 and *p* = 0.002, respectively). At the 8-week time point a significant improvement was maintained in the HD group only (*p* = 0.03) compared to the saline control group (Table 3).

**Table 3:** Micro CT results at 4- and 8-week time-points

Groups	Bone mineral density*	*p* value
Saline		
4 weeks	0.951 ± 0.0179	–
8 weeks	1.01 ± 0.852	–
Buffer + HA/TCP		
4 weeks	1.43 ± 0.116	**0.0001**
8 weeks	1.15 ± 0.301	0.72
Low-dose rhBMP-2 + HA/TCP		
4 weeks	1.50 ± 0.0101	**0.007**
8 weeks	1.06 ± 0.0223	0.90
High-dose rhBMP-2 + HA/TCP		
4 weeks	1.59 ± 0.217	**0.002**
8 weeks	1.37 ± 0.271	**0.03**

* Values represent ratio of BMD on operative side to that on contralateral non-operative side (mean ± standard deviation)

*p* values represent comparison to control group. *Bold* indicates statistical significance

**Fig. 3:**
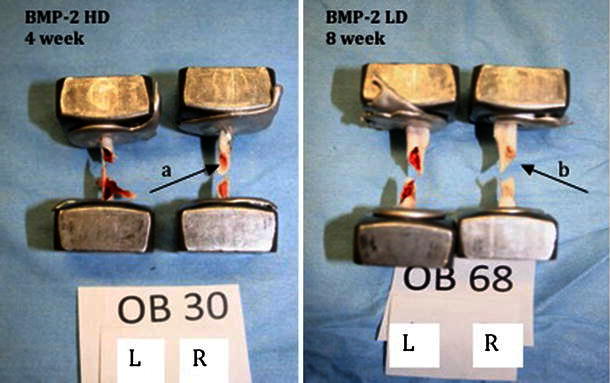
Spiral fractures resulting from torsional testing. Pictures show mid-diaphyseal spiral fractures. Note that the IM canals of the treated right femora appear *lighterred* (**a**) and *white* (**b**) due to the presence of HA/TCP carrier

The effect of locally delivered rhBMP-2 with a HA/TCP carrier on the biomechanical properties of osteoporotic bone was measured by torsional mechanical testing. As expected with intact femora, torsional testing to failure consistently resulted in mid-diaphyseal spiral fractures (Fig. 3). Although statistical analysis of general health parameters between treatment groups showed no significant differences, the inclusion criteria (based upon onset of T1DM) used in this study inherently created variability of age, weight, and weight change within treatment groups. Due to this variability, mechanical testing values were normalized to the contralateral limb, thus providing a more accurate assessment of the effects of rhBMP-2 treatment on biomechanical properties of the bone.

The treated femur values and normalized values for both time points showed no statistical differences between the four treatment groups in all categories except maximum torque to failure and peak shear (Tables 4, 5). While the statistical analysis lacked significance (*p* > 0.05), normalized data for both 4-week rhBMP-2-treated groups revealed strong trends toward dose-dependent improvement of peak torque, maximum torsional rigidity, maximum shear stress, and shear modulus (Table 4). Comparison of the combined rhBMP-2 groups and combined control groups demonstrated substantial differences close to statistical significance. Combined 4-week LD and HD rhBMP-2-treated groups showed 15 and 20 % increases in peak torque (*p* = 0.056) and torsional rigidity (*p* = 0.072), respectively, when compared to combined 4-week saline and HA/TCP groups (Table 6).

**Table 4:** 4- and 8-week post-treatment mechanical testing

Groups	Maximum torque to failure (N m)	*p* value	Torsional rigidity (N m/rad)	*p* value	Maximum shear stress (MPa)	*p* value	Shear modulus (MPa)	*p* value
Saline								
4 weeks (*n* = 5)	728 ± 84	0.89	62,969 ± 12,195	0.77	210 ± 26.29	0.29	7,940 ± 1,158.95	0.70
8 weeks (*n* = 8)	647 ± 149	**0.04**	63,836 ± 18,828	0.89	210 ± 46	0.22	9,239 ± 2,554	0.69
Buffer + HA/TCP								
4 weeks (*n* = 6)	754 ± 144		57,740 ± 17,801		245.64 ± 36		8,306 ± 2,153.17	
8 weeks (*n* = 6)	828 ± 70		63,889 ± 10,177		258 ± 33		8,608 ± 1,088	
Low-dose rhBMP-2 + HA/TCP								
4 weeks (*n* = 5)	789 ± 104		65,992 ± 5,806		253 ± 46		9,030 ± 936	
8 weeks (*n* = 6)	765 ± 105		59,259 ± 14,865		234 ± 35		7,930 ± 1,914	
High-dose rhBMP-2 + HA/TCP								
4 weeks (*n* = 6)	740 ± 174		63,948 ± 14,238		223 ± 45		8,461 ± 1,164.52	
8 weeks (*n* = 6)	805 ± 130		65,437 ± 12,384		244 ± 52		8,517 ± 1,976	

Treated femur values; one-way ANOVA comparing all four groups at 4 and 8 weeks (mean ± standard deviation)

*p* value in *bold* indicates statistical significance

**Table 5:** 4- and 8-week post-treatment mechanical testing (treated femur values normalized to normal (contralateral) side, one-way ANOVA to compare all four groups at 4 and 8 weeks)

Groups	Percent maximum torque to failure	*p* value	Percent torsional rigidity	*p* value	Percent maximum shear stress	*p* value	Percent shear modulus	*p* value
Saline								
4 weeks (*n* = 5)	107 ± 12	0.20	110 ± 29	0.12	100 ± 14	0.25	103 ± 29	0.53
8 weeks (*n* = 8)	83 ± 18	**0.01**	106 ± 25	0.85	82 ± 17	**0.02**	105 ± 34	0.73
Buffer + HA/TCP								
4 weeks (*n* = 6)	102 ± 17		85 ± 26		115 ± 21		95 ± 31	
8 weeks (*n* = 6)	117 ± 17		111 ± 18		106 ± 11		97 ± 15	
Low-dose rh-BMP-2 + HA/TCP								
4 weeks (*n* = 5)	116 ± 22*		118 ± 17*		117 ± 20*		115 ± 19*	
8 weeks (*n* = 6)	115 ± 24		98 ± 19		107 ± 22		89 ± 12	
High-dose rh-BMP-2 + HA/TCP								
4 weeks (*n* = 6)	123 ± 21*		116 ± 27*		124 ± 33*		118 ± 39*	
8 weeks (*n* = 6)	105 ± 19		102 ± 40		106 ± 12		100 ± 33	

Treated femur values normalized to untreated (contralateral) side; one-way ANOVA comparing all four groups at 4 and 8 weeks (mean ± standard deviation)

*p* value in *bold* indicates statistical significance

* Indicates a noticeable dose dependent trend in all four mechanical properties, although no statistical significance exists.

**Table 6:** 4-week mechanical testing

	Percent maximum peak torque	Percent torsional rigidity	Percent maximum shear stress	Percent shear modulus
Combined controls (*n* = 11)	104 ± 14	96 ± 30	108 ± 19	99 ± 0.287
rhBMP-2 (HD + LD) (*n* = 11)	120 ± 21	117 ± 22	121 ± 27	117 ± 30
	*p* = 0.056	*p* = 0.072	*p* = 0.224	*p* = 0.167

4-week normalized ratio of right:left femur; combined treated vs. combined untreated (mean ± standard deviation)

Normalized values from the 8-week LD rhBMP-2 group revealed a continued significant improvement in peak torque, and revealed significant improvement in peak shear (Table 5). Conversely, the normalized values for the 8-week HD rhBMP-2 group did not maintain the trend towards improved biomechanical properties seen in the 4-week HD rhBMP-2 group. The HA/TCP group, although not statistically significant, did perform better at 8 weeks than at 4 weeks in maximum torsional rigidity and exhibited comparable results to the 8-week LD rhBMP-2 group (Table 5).

## Discussion

To our knowledge, this study is the first to extend research on rhBMP-2 into a non-fracture DM-related osteoporosis model with HA/TCP as a carrier. Numerous studies have investigated locally delivered rhBMP-2 in fracture, segmental defect, and ectopic bone formation models but few have studied its effects on intact bone [14, 18].

Based upon the proven osteogenic effects of rhBMP-2 and the theory of local growth factor deficiencies in DM-related osteoporosis, it was hypothesized that local delivery of rhBMP-2 via a HA/TCP carrier would improve the bone mineral density and biomechanical properties of intact, DM-induced osteoporotic bone in the DM BB Wistar rat. Bone mineral density of both LD and HD rhBMP-2 groups at 4 weeks and of the HD rhBMP-2 group at 8 weeks was significantly improved compared to the saline control group. However, while a dose-dependent increasing trend in peak torque, torsional rigidity, shear stress, and shear modulus was noted in subsequent biomechanical testing, significance was not reached.

Since its discovery approximately 25 years ago, research has provided important insights into the role of BMPs and their effect on bone. Like most protein signaling substrates in the human body, their role is vast, diverse, and regulated in a complex manner, with both anabolic and catabolic effects. The BMP family, a sub-division of the TGF gene superfamily, exerts its effects on bone via several pathways, but in general by inducing the formation of extracellular matrix (cartilaginous scaffold), and later up- and down-regulating the promotion of osteoclasts and osteoblasts [22]. Often occurring via the promotion of angiogenesis, this pathway is affected by diabetes, and theoretically BMPs can improve bone rigidity and density via vascular regeneration [23–25]. Thus, delivering local BMP, even to intact bone, can help promote bone rigidity, and with an HA/TCP carrier a dose-dependent increase was noted. However, efficacy was lost after 8 weeks and this was likely due to the feedback control cited in the literature.

BMPs, in general, are only beneficial in specific dose ranges. Recent studies have demonstrated increased osteoclast activity and decreased anabolic effects in response to high-dose BMPs [26, 27]. Toth et al. [27] demonstrated concentration-dependent osteoclastic resorption of peri-implant bone associated with high levels of rhBMP-2. Furthermore, Vanketesh et al. hypothesized a noggin-mediated negative feedback control mechanism which decreased anabolic activity at high doses of rhBMP-4 [26]. These findings introduce valid questions concerning dosage. It is possible that HD rhBMP-2, which showed improved BMD at both 4- and 8-week time points, may cause decreased anabolic responses at longer time-points.

Interestingly, the 8-week HA/TCP group performed better than the 4-week HA/TCP group in both peak torque and torsional rigidity and revealed similar trends to the 8-week LD rhBMP-2 group in peak torque. While BMP has been shown to lose its effect in the short term, it is reasonable to attribute the improved rigidity in the long term to the osteoconductivity of the carrier [28–31] as well as the stimulatory effect of the intramedullary reaming [32–35].

Wang et al. [36] conducted the only other study in intact bone, albeit in an osteonecrotic model. Examining the controlled localized release of rhBMP-2 in regeneration of osteonecrotic bone, rhBMP-2 was delivered via poly(lactic-glycolic acid) (PLGA)-HA microspheres to the site of necrotic bone utilizing an in vivo mouse model. Wang et al. reported improved osteogenesis and neo-vascularization at 2 and 4 weeks in rhBMP-2-treated groups.

DM-related osteoporosis fracture and segmental defect models inherently create greater differences between treated and untreated groups. Because this study dealt with intact bone, the differences in biomechanical properties of the bone were not as drastic and therefore required larger sample sizes for significance. In fact, drastic increases in certain properties would be detrimental in that they may create an imbalance between the rigid and flexible properties of bone. The consistency of increased strength and rigidity in both extrinsic and intrinsic properties in the experimental groups at 4 weeks suggests balanced improvement in the quality of the bone.

However, despite its limitations, this study sheds important light on the effect of rhBMP on diabetes-induced osteoporotic bone. Adding to the literature of utilizing HA/TCP as an important carrier could have important, immediate clinical translations. While improving overall BMD, further studies should look at the potential effects on angiogenesis and subsequent strengthening in diabetic bone. Future research to expand upon this specific study should include additional histological analysis as well as increasing animal numbers in order to obtain statistical significance. Additional studies in humans could be performed later to determine the benefit of rhBMP-2 in patients who are at risk of developing osteoporotic fractures, especially in the setting of diabetes.
